# The urban-rural divide in complementary and alternative medicine use: a longitudinal study of 10,638 women

**DOI:** 10.1186/1472-6882-11-2

**Published:** 2011-01-06

**Authors:** Jon Adams, David Sibbritt, Chi-Wai Lui

**Affiliations:** 1 School of Population Health, University of Queensland, Brisbane, Queensland, Australia; 2 School of Medicine and Public Health, University of Newcastle, New South Wales, Australia

## Abstract

**Background:**

Research has identified women in rural and remote areas as higher users of complementary and alternative medicine (CAM) practitioners than their urban counterparts. However, we currently know little about what influences women's CAM consumption across the urban/rural divide. This paper analyses 10,638 women's CAM use across urban and rural Australia.

**Methods:**

Data for this research comes from Survey 5 of the Australian Longitudinal Study on Women's Health conducted in 2007. The participants were aged 56-61years. The health status and health service use of CAM users and non-users were compared using chi-square tests for categorical variables and t-tests for continuous variables.

**Results:**

Women who consulted a CAM practitioner varied significantly by place of residence: 28%, 32% and 30% for urban, rural and remote areas respectively (*P < .005*). CAM users tended to be more dissatisfied with conventional care than CAM non-users, but this was consistent across the 3 areas of residence. CAM users have higher percentages of most symptoms but the only rural/urban differences were for severe tiredness, night sweats, depression and anxiety. For diagnosed diseases, CAM users have higher percentages of most diagnoses but only hypertension and skin cancer were statistically significantly higher for rural and remote but not urban women (*P < .005*).

**Conclusions:**

In contrast to some recent claims, our analysis suggests the lack of access to and/or patient dissatisfaction with conventional health practitioners may not play a central role in explaining higher use of CAM by women in rural and remote areas when compared to women in urban areas.

## Background

The use of complementary and alternative medicine (CAM) has achieved mainstream status in Western countries over past decades [1-4] and surveys on patterns of CAM use reveal that such health seeking behavior is not confined to metropolitan settings. There is evidence that residents in rural and remote regions also employ a variety of treatments to complement their conventional care and to manage chronic health problems like diabetes and arthritis [5-13]. Previous surveys on patients of rural community centers or health clinics suggest prevalence rates for CAM use ranging from between 39% and 87% [6-12]. CAM is also found to be used by older rural adults as a common strategy for maintaining health and wellbeing (as distinct from treating specific health problems and conditions) [12-17].

What is more, research findings (especially from North America and Australia) show rural and remote populations as linked with higher CAM consumption relative to their urban counterparts [8,18-21]. A retrospective analysis of 237,500 claims data of two large US insurance companies in 2002 found that the proportion of claimants using chiropractors was higher among rural residents when compared to urban residents even though users of chiropractic in metropolitan areas made more chiropractic visits than users in non-urban areas [22]. In Australia, the PUC-CAM (Perspectives on the Use in Communities of Complementary and Alternative Medicine) study based on a survey of 459 residents in Victoria revealed significantly higher rural use of self-prescribed supplements, chiropractic and Bowen therapy than in urban areas [19,23]. A longitudinal analysis of mid-age Australian women's use of CAM also suggested that CAM consumption is higher in non-urban regions than in metropolitan areas [24,25].

Researchers have promoted a number of possible explanations for a higher rate of CAM use in rural and remote areas. These include limited access to health services and/or patient dissatisfaction with conventional health care services in non-urban regions, closer working ties between regional general practice and CAM provision, and stronger informal community networks in rural settings [11,26-30]. However, no research to date has empirically tested such propositions.

Given the popularity of CAM in rural and remote health, it is somewhat surprising that many studies have focused either solely on the use of CAM in metropolitan settings or CAM use in general populations and little is known about women's CAM consumption across the urban/non-urban divide [2,18,31-34]. In response, this paper - analyzing 10,638 women's CAM use across urban, rural and remote regions in Australia - provides the first step to fill this important research gap.

## Methods

### Sample

This research was conducted as part of the Australian Longitudinal Study on Women's Health (ALSWH) which was designed to investigate multiple factors affecting the health and well being of women over a 20-year period. Relevant ethical approval was gained from the Human Ethics Committee at the University of Queensland and University of Newcastle, Australia. Women in three age groups ("young" 18-23, "mid-age" 45-50 and "older" 70-75 years) were randomly selected from the national Medicare database [35]. The focus of this study is women from the mid-age cohort who have been surveyed five times over a ten year period (1996-2007) and their answers provide the data for longitudinal analysis. The baseline survey, survey 1 (n = 14,779), was conducted in 1996 and the respondents have been shown to be broadly representative of the national population of women in the target age groups [36]. Analyses for this study are restricted to the most recent survey, which was conducted in 2007 (n = 10,638).

### Area of residence

The postcode of usual residence for each woman in the ALSWH was used to allocate Rural, Remote and Metropolitan Areas (RRMA) score [37]. The RRMA index reflects distance from both service centers and from other people [38].

### Measures of health service use

The women were asked about their frequency of use in the previous twelve months of a general practitioner (GP) and a specialist doctor. In addition, they were asked if they had consulted with a hospital doctor or a CAM practitioner (i.e. chiropractor, massage therapist, acupuncturist, naturopath/herbalist, other CAM practitioner) in the previous twelve months.

### Measures of health status

Women were asked how often they had sought help for a list of 13 symptoms (such as back pain, severe tiredness, depression, anxiety) in the previous twelve months. Women were also asked whether they had been diagnosed with any of 12 chronic medical conditions (such as diabetes, arthritis, heart disease, hypertension, breast cancer).

### Rating of conventional health care providers

The women were asked to rate their level of satisfaction with various aspects of conventional health care providers (such as access to a female GP, hours when a GP is available, outcomes of medical care). Each aspect was rated via a 5-point Likert scale, where 1 = excellent and 5 = poor.

### Statistical Analyses

Student t-tests were used to compare the binary CAM user status variable against continuous variables. Chi-square tests were used to compare the binary CAM user status variable against categorical variables. In response to the large sample size and multiple comparisons, a p-value < 0.005 was adopted for the level of statistical significance in all statistical comparisons. All analyses were conducted using the statistical software SAS 9.1.

## Results

There were 10,638 women who completed and returned the questionnaire, of which 40% were living in urban areas, 56% in rural areas and 4% in remote areas. Among these women, 30% indicated that they had consulted with a CAM practitioner in the previous 12 months. The percentage of women who consulted a CAM practitioner varied by place of residence: 28%, 32% and 30% for urban, rural and remote areas respectively. This association was statistically significant (*P < .005*).

Table 1 shows that overall CAM users tend to consult with a GP more frequently than CAM non-users (*P < .005*). This pattern can be seen separately for the 3 areas of residence, although the association is only statistically significant (*P < .005*) for urban and rural areas. Similarly, CAM users tend to consult with a specialist doctor more frequently than CAM non-users within the 3 areas of residence and overall (*P < .005*). There is no statistically significant association between CAM user status and consultation with a hospital doctor overall or within the 3 areas of residence.

**Table 1:** Consultations with conventional health care providers by CAM user status (consulted with a CAM practitioner or not)

		Urban		Rural		Remote	
		
		CAM non-user	CAM user	CAM non-user	CAM user	CAM non-user	CAM user
Consultations		(n = 2,960) %	(n = 1,157) %	(n = 4,011) %	(n = 1,866) %	(n = 308) %	(n = 131) %
**GP ^■ 1 2^**	0	6	5	7	5	9	8
	1-2	34	27	36	32	36	31
	3-4	31	32	29	28	28	32
	5-6	16	17	15	18	14	16
	7-12	9	13	9	11	9	10
	13+	4	6	4	6	4	3

**Hospital Doctor**	0	83	80	82	80	80	76
	1-2	13	15	15	16	14	19
	3+	4	5	3	4	6	5

**Specialist Doctor ^■ 1 2 3^**	0	51	44	57	52	63	48
	1-2	32	34	30	32	25	39
	3-4	11	13	8	10	8	8
	5-6	3	5	3	3	1	1
	7+	3	4	2	3	3	4

*Note: chi-square tests used to test for statistically significant associations*.

^■ ^Statistically significant association for CAM user status (i.e. ignoring place of residence) (*P < .005*)

^1 ^Statistically significant association for urban residents (*P < .005*)

^2 ^Statistically significant association for rural residents (*P < .005*)

^3 ^Statistically significant association for remote residents (*P < .05*)

The relationship between the rating of various aspects of conventional health care provision and CAM user status is shown in Table 2. In general, CAM users were more dissatisfied with the outcomes of their medical care than CAM non-users (*P < .005*). This was a consistent pattern across the 3 areas of residence, although it was only statistically significant for women from urban and rural areas (*P < .005*). Furthermore, CAM users were more dissatisfied with the hours when a GP was available, the ease of seeing a GP of their choice, and the waiting time to get a GP appointment than CAM non-users (*P < .005*). There were consistent patterns across the 3 areas of residence for all of these aspects, although they were only statistically significant for women from urban areas (*P < .00*5). CAM users in urban areas only were also more dissatisfied with access to a medical specialist if needed compared to CAM non-users (*P < .005*). There were no statistically significant associations, either overall or for separate areas of residence, between CAM user status and access to a female GP.

**Table 2:** Rating of conventional health care providers by CAM user status (consulted with a CAM practitioner or not)

	Urban				Rural				Remote			
	
Level of Satisfaction (1 = excellent ... 5 = poor)	CAM non-user		CAM user		CAM non-user		CAM user		CAM non-user		CAM user	
	(n = 2,960)		(n = 1,157)		(n = 4,011)		(n = 1,866)		(n = 308)		(n = 131)	
	mean	SD	mean	SD	mean	SD	mean	SD	mean	SD	mean	SD
**Access to a medical specialist if needed**^1^	1.93	0.95	2.02	1.03	2.50	1.15	2.48	1.13	3.11	1.28	3.14	1.31

**Access to a female GP**	2.24	1.22	2.30	1.25	2.76	1.37	2.74	1.35	3.39	1.40	3.44	1.39

**Hours when a GP is available**^■ 1^	2.65	1.10	2.80	1.09	2.90	1.14	2.97	1.14	3.22	1.12	3.34	1.20

**Number of GPs you have to choose from**^■ 1^	2.50	1.13	2.62	1.14	2.94	1.25	2.97	1.23	3.67	1.22	3.67	1.27

**Ease of seeing GP of your choice**^■ 1^	2.58	1.18	2.75	1.19	2.97	1.27	3.04	1.26	3.32	1.31	3.50	1.32

**How long you wait to get a GP appointment**^■ 1^	2.74	1.14	2.87	1.13	3.12	1.20	3.18	1.19	3.35	1.21	3.54	1.12

**The outcomes of your medical care**^■ 1 2^ **(how much you are helped)**	2.27	0.96	2.41	0.99	2.45	0.98	2.54	0.99	2.67	0.96	2.81	1.03

*Note: Student t-tests used to test for statistically significant differences*.

^■ ^Statistically significant association for CAM user status (i.e. ignoring place of residence) (*P < .005*)

^1 ^Statistically significant association for urban residents (*P < .005*)

^2 ^Statistically significant association for rural residents (*P < .005*)

^3 ^Statistically significant association for remote residents (*P < .05*)

Table 3 shows the association between the symptoms that women sought help for and consultations with a CAM practitioner. Overall, CAM users were significantly more likely than non-users to seek help for severe tiredness (*P < .005*). This was a consistent pattern across all the 3 areas of residence, although it was only statistically significant for women from rural and remote areas (*P < .005*). CAM users were also significantly more likely than non-users to seek help for night sweats and anxiety (*P < .005*). There were consistent patterns across the 3 areas of residence for these two aspects, although they were only statistically significant for women from rural areas (*P < .005*). A greater percentage of CAM users than non-users sought help for depression across the 3 areas of residence, but this was only statistically significant for women from remote areas (*P < .005*). The analysis also demonstrates that CAM users were significantly more likely to seek help for back pain than non-users (*P < .005*). This was a consistent and statistically significant pattern across the 3 areas of residence (*P < .005*). In addition, CAM users were significantly more likely to seek help for indigestion or heartburn, headaches or migraines, stiff or painful joints, urine that burns or stings, hot flushes (*P < .005*) than non-users. There were consistent patterns across the 3 areas of residence for all of these aspects, although they were only statistically significant for women from urban and rural areas (*P < .005*). Finally, CAM users were significantly more likely than non-users to seek help for allergies or hayfever or sinusitis (*P < .005*). This was a consistent and statistically significant pattern across the 3 areas of residence, although it was only statistically significant for women from urban areas (*P < .005*). There were no statistically significant associations between CAM user status and breathing difficulties or chest pain.

**Table 3:** Sought help for symptoms by CAM user status (consulted with a CAM practitioner or not)

		Urban		Rural		Remote	
		
Sought help for the following symptoms:		CAM non-user	CAM user	CAM non-user	CAM user	CAM non-user	CAM user
		(n = 2,960)	(n = 1,157)	(n = 4,011)	(n = 1,866)	(n = 308)	(n = 131)
**Allergies, hayfever, sinusitis**^■ 1^	% yes	13	19	13	16	13	16

**Breathing difficulties**	% yes	7	9	8	9	7	6

**Indigestion or heartburn**^■ 1 2^	% yes	9	13	9	13	10	11

**Chest pain**	% yes	6	7	6	6	6	8

**Headaches or migraines**^■ 1 2^	% yes	6	9	7	10	6	9

**Severe tiredness**^■ 2 3^	% yes	6	8	6	9	5	10

**Stiff or painful joints**^■ 1 2^	% yes	15	26	18	25	15	18

**Back pain**^■ 1 2 3^	% yes	12	31	12	29	11	29

**Urine that burns or stings**^■ 1 2^	% yes	4	7	4	7	6	3

**Hot flushes**^■ 1 2^	% yes	6	10	6	10	5	5

**Night sweats**^■ 2^	% yes	5	7	4	7	4	5

**Depression**^3^	% yes	8	9	8	9	4	9

**Anxiety**^■ 2^	% yes	7	9	6	9	4	6

*Note: chi-square tests used to test for statistically significant associations*.

^■ ^Statistically significant association for CAM user status (i.e. ignoring place of residence) (*P < .005*)

^1 ^Statistically significant association for urban residents (*P < .005*)

^2 ^Statistically significant association for rural residents (*P < .005*)

^3 ^Statistically significant association for remote residents (*P < .05*)

Table 4 shows the association between the diseases that women have been diagnosed for and consultations with a CAM practitioner. Overall, CAM users were more likely than non-users to have osteoporosis (*P < .005*). This was a consistent, statistically significant pattern across the 3 areas of residence (*P < .005*). CAM users were also more likely to have asthma and bronchitis/emphysema (*P < .005*). There were consistent patterns across the 3 areas of residence for these two diagnoses, although they were not statistically significant for the separate areas of residence. The analysis shows that CAM users were more likely to have arthritis (*P < .005*). This was a consistent pattern across the 3 areas of residence, although it was only statistically significant for women from urban and rural areas (*P < .005*). CAM users were also more likely to have low iron levels (*P < .005*). This was a consistent pattern across the 3 areas of residence, although it was only statistically significant for women from urban areas (*P < .005*). In addition, CAM users were more likely to have hypertension (*P < .005*) than non-users. This was a consistent pattern across the 3 areas of residence, although it was only statistically significant for women from rural areas (*P < .005*). Finally, CAM users were more likely than non-users to have skin cancer (*P < .005*). This was a consistent pattern across the 3 areas of residence, although it was only statistically significant for women from remote areas (*P < .005*). For all other cancers and for diabetes and heart disease there were no statistically significant associations with CAM user status.

**Table 4:** Diagnoses by CAM user status (consulted with a CAM practitioner or not)

		Urban		Rural		Remote	
		
Diagnoses		CAM non-user	CAM user	CAM non-user	CAM user	CAM non-user	CAM user
		(n = 2,960)	(n = 1,157)	(n = 4,011)	(n = 1,866)	(n = 308)	(n = 131)
**Diabetes**	% yes	26	26	27	26	31	24

**Arthritis**^■ 1 2^	% yes	34	40	35	41	33	36

**Heart Disease**	% yes	6	6	7	7	8	7

**Hypertension**^■ 2^	% yes	32	32	38	33	41	32

**Low iron level**^■ 1^	% yes	30	36	28	32	26	34

**Asthma**^■^	% yes	19	21	19	21	18	20

**Bronchitis/emphysema**^■^	% yes	18	20	17	19	13	18

**Osteoporosis**^■ 1 2 3^	% yes	11	15	9	11	5	11

**Breast cancer**	% yes	5	6	5	5	3	4

**Cervical cancer**	% yes	3	3	3	3	2	2

**Skin cancer**^■ 3^	% yes	20	21	21	24	19	33

**Other cancer**	% yes	4	5	5	5	4	3

*Note: chi-square tests used to test for statistically significant associations*.

^■ ^Statistically significant association for CAM user status (i.e. ignoring place of residence) (*P < .005*)

^1 ^Statistically significant association for urban residents (*P < .005*)

^2 ^Statistically significant association for rural residents (*P < .005*)

^3 ^Statistically significant association for remote residents (*P < .05*)

## Discussion

In line with previous research findings [8,18-22,24,25], our study shows that a significantly higher percentage of women from rural (32%) and remote (30%) areas consult with a CAM practitioner when compared to those women in urban areas (28%). This indicates the use of CAM may play a distinctive role in the health management of women in remote or geographically isolated locations. The levels of consumption identified across the longitudinal analysis also highlight the importance for rural conventional providers to enquire about the use of complementary and alternative therapies or modalities with the people in their care.

Findings from this study also suggest that, in contrast to some recent claims [11,26-29], the lack of access to and/or patient dissatisfaction with conventional health practitioners may not play an important role in explaining the higher use of CAM in non-urban regions when compared to metropolitan areas. In addition, although our study shows female CAM users tend to have a higher percentage of health symptoms/diagnoses of chronic illnesses when compared to non-CAM users, such differences were largely consistent across urban and rural/remote areas. This suggests health status may not be an important contributing factor to differences in CAM practitioner use across the urban/non-urban divide.

When considered together, these findings suggest a need to investigate possible influences beyond user attributes or values to account for women's high use of CAM practitioners in rural and remote settings. This includes examination of the influences of informal community networks, traditional cultural beliefs and closer ties or referral between regional GPs and CAM practitioners in rural areas. Researchers are also recommended to pay attention to differences between the types of CAM used by women across geographical location.

In interpreting our study findings it is important to remain mindful of two design limitations. The study defined CAM use as consultation with an alternative health practitioner and did not include the use of self-prescribed CAM medications or self-directed CAM practices. Previous research has identified high prevalence of such CAM self-care [5,13,19] and this definition may have led to the under-reporting of CAM use in the study. This limitation is the result of our study employing a secondary analysis of a larger longitudinal study that was not specifically designed to examine CAM use. In addition, the use of a restricted list of CAM practitioners may also have skewed the study findings even though an 'other' category was included.

In addition, a drawback of conducting statistical analyses on studies with large sample sizes is potential for analyses to be statistically over-powered and hence an increased risk of making a Type I error. That is, a trivial difference may be mistakenly understood to be a significant one. We have attempted to overcome this potential problem by setting the level of statistical significance at α = 0.005 instead of the conventional α = 0.05. However, caution should still be used when interpreting the findings, particularly for comparisons made on the urban and rural groups.

## Conclusions

Given the evidence supporting an urban/rural distinction in women's CAM use, it is important that we further investigate and understand the reasons for such geographical differences in CAM consumption. Future research is required to examine wider social and interpersonal factors as well as characteristics of CAM providers in an attempt to help explain the high use of CAM in non-urban areas. Such work may include examination of the availability of CAM practitioners [39,40], the interface and/or referral between conventional and CAM practitioners [30], as well as the nature of the patient-practitioner relationship across conventional and CAM practice lines in rural and remote settings [7,9,41,42]. This research will provide insights of importance to rural and remote practitioners, patients, health managers and policy-makers in their efforts to provide effective rural and remote health care services and provision.

## Competing interests

The authors declare that they have no competing interests.

## Authors' contributions

All authors devised the study and helped to conceptualize ideas, interpret findings, manuscript writing and reviewing drafts of the article. DS undertook data analysis and JA and CWL contributed to the interpretation of the data.

## Pre-publication history

The pre-publication history for this paper can be accessed here:

http://www.biomedcentral.com/1472-6882/11/2/prepub
